# “No Fair!”: Children’s Perceptions of Fairness in Merit-Based Distributions

**DOI:** 10.3390/bs16040617

**Published:** 2026-04-21

**Authors:** Meltem Yucel, Madeline Brence, Amrisha Vaish

**Affiliations:** 1 Department of Psychology, Michigan State University, East Lansing, MI 48824, USA; 2 Department of Psychology, University of Virginia, Charlottesville, VA 22903, USAvaish@virginia.edu (A.V.)

**Keywords:** conventional norms, fairness, inequality, merit, moral development, moral norms

## Abstract

Recent research by Yucel and colleagues suggests that children perceive equality-based fairness violations (resources being distributed unequally) as less serious than prototypical moral harms, but that making the harmful consequences of unfairness salient shifts these judgments toward the moral domain. We examined whether merit-based fairness violations (someone receiving less than they earned) would similarly shift judgments toward the moral domain by making the injustice more salient. Replicating prior work, 4-year-old children (*N* = 62) rated prototypical moral violations as significantly more severe than equality-based fairness violations, which were rated as similar in severity to conventional violations. Contrary to predictions, merit-based fairness violations also showed this pattern: They were judged as less severe than prototypical moral violations and similarly severe as both equality-based fairness violations and conventional violations. Children also did not consistently group either type of fairness violation with moral or conventional violations. These findings contribute to a growing body of evidence that children’s (and adults’) perceptions of fairness—whether equality-based or merit-based—are more nuanced than previously thought and that unfairness may not spontaneously be treated like other, more prototypical moral norm violations.

## 1. Introduction

Fairness is a cornerstone of human social life. People care deeply about whether resources and opportunities are distributed justly and react with frustration and disappointment when they are not. This concern is reflected in ongoing social debates—from controversies over equal pay (such as recently between the U.S. Men’s and Women’s National Soccer Teams) to questions about who deserves what and why (Hernandez, 2022). This sensitivity to unfairness is not limited to adults; even young children attend and react to unequal outcomes (Baumard et al., 2012; Dietze & Craig, 2021; Kanngiesser & Warneken, 2012; Schäfer et al., 2023; Sloane et al., 2012). Yet it remains unclear how children *conceptualize* such situations. When resources are distributed unfairly, do they see this as a moral violation (akin to hitting someone) or as a less serious violation of social rules or expectations (akin to wearing pajamas to school)? The present work aims to clarify how fairness violations fit into the developing landscape of moral judgment.

In addressing this question, we focus on how resources are allocated between individuals (i.e., distributional fairness). This represents one form of fairness; people also care about procedural fairness (whether decisions are made through fair processes) and retributive fairness (how wrongdoing is punished or rectified) (Acar & Sivis, 2023; Deutsch, 1975; Dunham et al., 2018; Rawls, 1971). Distributional fairness (henceforth simply referred to as “fairness”) has been the subject of extensive developmental research with children and thus provides an ideal context for exploring how children perceive fairness norms early in development.

Children’s understanding of equality-based fairness emerges early. Within the first year, infants expect resources to be divided equally (Schmidt & Sommerville, 2011; Sommerville et al., 2013). Starting around 3 years of age, children endorse equal sharing (that resources should be evenly distributed) and even enforce fairness norms on others (Rakoczy et al., 2016; Smith et al., 2013). Yet at the same time, young children often fail to share equally themselves (Fehr et al., 2008; Smith et al., 2013). Thus, expectations of equality-based fair distributions emerge early, and both understanding and behavior become more robust with age.

To understand children’s perceptions of fairness violations, we situate them within the broader framework of moral and conventional norms as conceptualized by Social Domain Theory (SDT) (Killen & Smetana, 2015; Nucci & Turiel, 1978). By age 3, children begin to distinguish *moral norms*, or norms concerning others’ welfare or how to treat others (such as not hitting people or destroying their property), from *conventional norms*, or norms that help coordinate institutions or social interactions (such as abiding by dress codes or playing a game correctly) (Nucci & Turiel, 1978; Yucel et al., 2020). Children and adults not only evaluate these two norm types differently, but also show distinct physiological responses to their violation, with greater arousal to moral than conventional violations (Kassecker et al., 2023; Yucel et al., 2020).

Within this framework, fairness norms, which concern the distribution of resources, have often been assumed to belong to the moral domain and are frequently grouped with other prototypical moral norms involving physical harm (Ball et al., 2017; Noh, 2020; Smetana, 1981). Recent work with children and adults, however, challenges this assumption. Specifically, studies examining equality-based unfairness reveal that it occupies an ambiguous position between moral and conventional violations. For example, Yucel et al. (2022) found that children’s judgments of equality-based unfairness do not align straightforwardly with prototypical moral violations: Although 6- and 8-year-olds viewed unfairness (e.g., one child taking more than another child) as more serious than conventional violations (e.g., wearing pajamas to school), they still judged it as less serious than harm-based moral violations (e.g., hitting). Four-year-olds judged unfairness to be about as serious as conventional violations, and both to be less serious than harm-based moral violations. Moreover, when asked to group equality-based unfairness with harm-based moral violations versus conventional violations, most 6-year-olds grouped unfairness with the moral violations, but 4- and 8-year-olds showed no consistent pattern. Yucel and Vaish (2026) replicated this overall pattern with 4-year-olds as well as adults. Consistent with this, Deutchman et al. (2025) found that adults perceived fairness and conventional behaviors as more alike, and distinct from harm-based behaviors.

Together, these findings suggest that equality-based fairness norms may be perceived more ambiguously within the moral-conventional landscape than previously thought. One possible explanation for this ambiguity is that, unlike prototypical moral violations (e.g., hitting, pushing), whose harm is direct and immediately visible, the harm caused by fairness violations is often indirect and less perceptible. This may make it difficult for young children to recognize unfairness as fundamentally harmful, leading them to treat it more like a conventional norm whose violation is undesirable but not obviously harmful (Ball et al., 2017; Rochat et al., 2009; Yucel et al., 2022).

We may therefore predict that making the harm caused by fairness more explicit should shift how fairness violations are perceived. Indeed, Yucel and Vaish (2026) found that both children’s and adults’ evaluations of equality-based unfairness shifted when the harmful consequences of unfairness were made salient. Unfairness was judged as a more severe violation—and thus closer in seriousness to prototypical moral violations—when its harmful consequences were highlighted than when they were not. Perceptions of fairness violations can therefore be shifted in a more “moral” direction when the harm or wrongness of an unfair act is made explicit.

This malleability motivates the present study’s focus on a specific type of fairness violation that may inherently convey harm or wrongness: merit-based unfairness. The *merit principle* holds that resources should be distributed proportional to effort or performance (Deutsch, 1975). Much like equality-based fairness, by 3 years of age, children begin to take effort into account when distributing resources (Baumard et al., 2012; Hamann et al., 2011; Kanngiesser & Warneken, 2012), and this ability becomes more robust with age (see Pesowski et al., 2022; Rizzo & Killen, 2020). Parents (at least in the U.S.) also explicitly communicate meritocratic beliefs to their children (Kinnard et al., 2025). Notably, the moral weight of merit-based fairness violations may itself be culturally contingent: Cross-cultural work shows variability in children’s responses to both equality- and merit-based unfairness, with children from Western societies placing greater emphasis on merit than those from egalitarian societies (Blake et al., 2015; Engelmann et al., 2021; Schäfer et al., 2015). Thus, whether fairness violations—and merit-based violations in particular—register as moral or conventional transgressions may vary across sociocultural contexts. The present study focuses on a U.S. sample, where meritocratic values are actively socialized (Kinnard et al., 2025), yet how children in this context conceptualize merit-based violations—particularly in relation to moral versus conventional norms—remains unclear.

We propose that merit-based violations may be perceived differently than simple equality-based violations because merit-based violations may make the harm more apparent. Unlike simple inequality (when one individual takes more than another without additional context), merit-based violations explicitly involve someone being denied what they have earned or deserve, which may be perceived as more clearly harmful or wrong and therefore as a more severe violation. If making harm salient increases the severity of equality-based unfairness, then merit information may operate similarly: Rather than requiring harmful consequences to be highlighted (as in Yucel & Vaish, 2026), the harm or injustice may be inherent in the violation structure itself, helping an observer see that the outcome is not merely unequal but a moral wrong. Accordingly, the present study asked whether children treat merit-based fairness violations as more severe and “moral-like” than simple equality violations and more similar in severity to harm-based moral violations.

### Current Investigation

In the present research, we investigated how 4-year-old children in the U.S. conceptualize merit-based fairness violations compared to equality-based violations and prototypical moral and conventional violations. We focused on 4-year-olds because prior work shows that by this age, children reliably judge moral violations as more serious than conventional violations but judge equality-based fairness violations as no more serious than conventional violations (Yucel et al., 2022; Yucel & Vaish, 2026). This leaves the greatest potential for merit information to shift seriousness evaluations upward toward the moral domain. Importantly, such upward movement is achievable at this age, as evident when the harmful consequences of unfairness were made more salient (Yucel & Vaish, 2026). Thus, if merit information helps highlight the injustice of unequal outcomes, children may judge merit-based violations as more severe and more moral-like than equality-based violations.

Our study had three aims. First, we aimed to replicate Yucel et al.’s (2022) and Yucel and Vaish’s (2026) findings regarding simple equality-based unfairness. Following this prior work, we presented children with scenarios involving moral, conventional, and equality-based fairness violations, as well as control scenarios. We asked children to evaluate the seriousness of these actions (act evaluation) and whether their permissibility depended on authority approval (authority contingency), and to categorize them with prototypical moral or conventional violations. We included the authority contingency measure as it is one core diagnostic within Social Domain Theory that distinguishes moral (authority- and rule-independent) violations from conventional (authority- and rule-dependent) violations (Killen & Smetana, 2015; Turiel, 2022). Notably, Yucel et al.’s (2022) Study 1 did not find evidence of rule-independence for equality-based unfairness (and their Study 2 did not include this measure). We expanded our assessment to include authority-independence, testing whether children would treat equality-based unfairness as authority-independent, as SDT would predict if fairness is a moral concern, or instead resemble Yucel et al.’s Study 1 pattern, suggesting that equality-based unfairness remains ambiguous in this criterion.

Second, with a separate group of children, we introduced parallel scenarios involving merit-based unfairness, in which one child took more resources than another child despite equal effort. This manipulation was designed to make salient the disadvantaged character’s investment and the implied entitlement to a fair share, thereby strengthening the seriousness of the unequal outcome in a way that resembles making the harm salient (Yucel & Vaish, 2026). Children evaluated the seriousness of these merit-based violations and categorized them with either moral or conventional violations. We examined whether emphasizing effort would lead children to view merit-based unfairness as more serious and more closely aligned with moral violations than equality-based violations.

Finally, as an exploratory question, we examined children’s justifications for their categorization of the fairness violations with moral or conventional violations (as in Yucel et al., 2022; Yucel & Vaish, 2026). We assessed whether children would appeal more to normative reasons (e.g., invoking rules, obligations, or rights), emotional reasons (e.g., referencing how someone feels), or both. These exploratory analyses provide a richer picture of the reasoning underlying children’s evaluations of fairness norms and violations.

## 2. Materials and Methods

### 2.1. Participants

Sixty-two 4-year-olds participated in a single 20 min session, in a between-subjects design with two conditions. Thirty-two 4-year-olds participated in the Merit condition (*M* = 54.58 months, *SD* = 3.70 months, range = 46.37–59.8 months; 16 girls, 16 boys), and 30 4-year-olds participated in the Equality (i.e., simple inequality) condition (*M* = 54.80 months, *SD* = 3.88 months, range = 48.13–60.8 months; 18 girls, 12 boys). Across the two conditions, 8 additional 4-year-old children were tested but excluded due to the child not wanting to participate in the study (*n* = 1), parents interfering and leading their children’s answers (*n* = 1), children being distracted and not engaging with the task (*n* = 4), study not displaying correctly (*n* = 1), and failure to pass memory checks (*n* = 1). The sample size was determined based on prior work (Yucel et al., 2022; Yucel & Vaish, 2026). Both procedures were approved by the University of Virginia’s Human Research Ethics committee, and parents consented to their children’s participation.

### 2.2. Procedure

The protocol for this study was adapted from Yucel et al. (2022) and Yucel and Vaish (2026). As in Yucel and Vaish (2026), the study was conducted on Qualtrics (Provo, UT, USA) with a researcher present to moderate the study over videoconferencing software (Zoom Video Communications, Inc., 2020), and we prerecorded all aspects of the study to increase experimental control and avoid any potential connectivity problems. The experimenter remained quiet throughout the study and only stepped in to answer the child’s questions or to remind the parent not to interfere with the study.

The Merit and Equality conditions differed in two ways. First, children in the Merit condition were presented with merit-based inequality scenarios whereas children in the Equality condition were presented with simple inequality scenarios without merit context or emphasis. Second, only children in the Equality condition were asked Authority Contingency questions. We included this measure in the Equality condition to obtain a fuller picture of how children conceptualize simple inequality, beyond severity and categorization alone, and to assess whether children treat simple equality norms as authority-independent (consistent with the moral domain) or as more authority-dependent (consistent with the conventional domain). We did not administer Authority Contingency questions in the Merit condition because our primary aim in that condition was to test whether making merit salient shifts children’s severity evaluations relative to the well-established Equality pattern.

#### 2.2.1. Practice Phase

**Figure 1:**
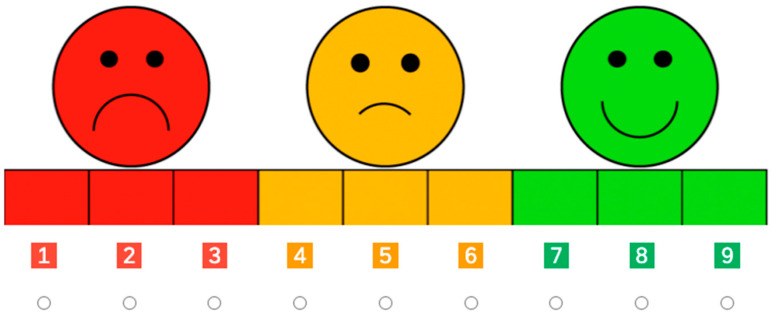
The response scale ranged from 1 (very, very bad) to 9 (very, very nice).

Participants were first familiarized with a 9-point response scale that has previously been used with 4-year-olds in both in-person and online settings (Yucel et al., 2022; Yucel & Vaish, 2026). This scale ranged from 1 (*very, very bad*) to 9 (*very, very nice*) (see Figure 1). Children were explicitly taught anchor and intermediate points: 1 (“very, very bad”), 3 (“very bad”), 4 (“bad”), 6 (“a little bit bad”), 7 (“a little bit nice”), and 9 (“very, very nice”), with the video recording emphasizing that the scale increased in positivity from left (red) to right (green). Unlabeled points (2, 5, 8) were described as falling between these anchors. To ensure understanding, children answered practice questions (e.g., identifying where to point for “very, very nice” or “a little bit bad”) and received feedback until they could reliably use the scale. Once children were sufficiently familiar with the scale, the test phase began.

#### 2.2.2. Test Phase

In a fully randomized order, children saw a total of 16 videos depicting three sets of violations (4 Moral, 4 Fairness, and 4 Conventional) and one set of control actions (4 Control). The two conditions differed in the type of fairness violations they were shown. The Moral, Conventional, and Control scenarios were identical across the Equality and Merit conditions. The conditions differed only in the Fairness scenarios described below.

The Moral vignettes depicted instances of physical or property-related harm, including hitting, pushing, pulling another child’s hair, and tearing another child’s picture (e.g., “This child hit this child.”). The Conventional vignettes depicted classroom or social norm violations, such as failing to put away a toy during snack time, wearing pajamas to school, sitting in the wrong place, and walking away during story time (e.g., “This child wore pajamas to school.”). The Control vignettes depicted everyday behaviors that were neutral or positive in their nature, including playing at the playground, saying “thank you” after receiving a toy, asking permission to play with a toy, and sitting down to eat with another child (e.g., “This child played at the playground.”).

The Fairness vignettes depicted unequal distributions in which one child took more resources than another. The two conditions differed in how these violations were framed. In the Equality condition, children viewed Fairness-Equality scenarios that described only the unequal taking action (e.g., “This child took more chalk than this child.”). This matched the fairness stimuli in prior work (Yucel et al., 2022; Yucel & Vaish, 2026). In the Merit condition, children viewed Fairness-Merit scenarios that presented the same unequal outcomes but first highlighted that the two children had contributed equally to gathering the resources before describing the unequal taking (e.g., “These children both helped the same amount to bring the chalk outside. During recess, this child took more chalk than this child.”). Thus, across conditions, the fairness scenarios were tightly matched on the unequal outcome, with the Merit condition adding contextual information intended to emphasize equal effort prior to the unfair distribution.

The full text of the procedure and all scenarios is provided in Appendix A.

##### Act Evaluation (Both Conditions)

After hearing each scenario, children were asked to evaluate the action using the 9-point scale (e.g., “How nice or bad is that?” see Figure 1). Once children had evaluated all 16 actions, those in the Merit condition moved directly to the Forced-Choice Categorization task whereas those in the Equality condition moved first to the Authority Contingency task before moving to the Forced-Choice Categorization.

##### Authority Contingency (Equality Condition Only)

In the Equality condition, for all scenarios (Moral, Conventional, Fairness, and Control), we assessed whether children’s evaluations were contingent on authority approval. Specifically, if a child evaluated any of the scenarios as bad in their Act Evaluation, the pre-recorded audio clip asked, “If your teacher said it was okay to do this, would it be okay or not okay?”

##### Forced-Choice Categorization (Both Conditions)

**Figure 2:**
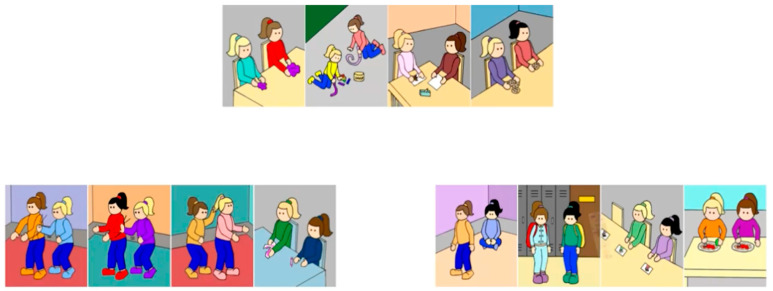
A still frame from the Forced-Choice Categorization task video. In this example, Moral scenarios are presented on the left and conventional scenarios are presented on the right side of the screen (location randomized across participants). Fairness scenarios are always presented at the top of the screen.

Using videos, children were asked whether the Fairness scenarios they had seen (either Fairness-Merit or Fairness-Equality) belonged with the set of four Moral violations or Conventional violations. Note that similar to previous studies (Yucel et al., 2022; Yucel & Vaish, 2026), neither the experimenter nor the video ever used the terms *moral*, *conventional*, *fairness*, *merit*, or *violation*; instead, the grouping question was presented visually, by showing the pictures of the four Moral violations on one side of the screen and four Conventional violations on the other side of the screen (location randomized), and asking which group the pictures of the four Fairness violations belonged with (see Figure 2). Also note that the child made one choice for the entire group of Fairness scenarios rather than evaluating each separately. Once the child picked either the Moral or Conventional group for the four Fairness pictures, the experimenter asked the child why they had picked that group (justification question).

### 2.3. Coding and Coder Agreement

Two researchers transcribed and coded children’s justifications for their Forced-Choice Categorization according to the coding scheme used in prior work (Yucel et al., 2022; Yucel & Vaish, 2026). There were four coding categories: (1) normative (comments about whether the actions were right/wrong or good/bad), (2) emotional (comments referring to kindness or explicit reference to emotions such as happiness or hurt feelings), (3) normative and emotional (a combination of both normative and emotional elements), or (4) irrelevant responses or no justification (see Table A1 in Appendix B for details of the coding scheme). The inter-rater reliability was excellent for both conditions (*κ_Equality_* = 0.94, *κ_Merit_* = 0.93).

### 2.4. Analytic Approach

All analyses were conducted in R version 4.5.2 (R Core Team, 2025).

For Act Evaluation, given the repeated-measures design and non-normal act-evaluation data for children in both conditions (*p*s < 0.001), we fit linear mixed-effects models to examine whether emphasizing merit changed children’s evaluations of fairness norms. Retaining all item-level responses (four trials per norm type per child) allowed the models to handle missingness and capture person-level heterogeneity. The fixed-effects structure included Norm Type (Moral, Fairness, Conventional, Control), Condition (Merit, Equality), and their interaction. Participant ID was entered as a random intercept in the model. Linear mixed-effects logistic regression models were analyzed using the *lmer* function, whereas generalized mixed-effects logistic regression models were analyzed using the *glmer* function from the *lme4* package in R (Bates et al., 2015). For within and across condition comparisons, we estimated marginal means using the *emmeans* package in R (Lenth & Piaskowski, 2025) and made multiple-comparison adjustments using Holm corrections (Holm, 1978). Our analytic approach was similar to Yucel and Vaish (2026).

For Authority Contingency (Equality condition only), which was binary (0 = not okay, 1 = okay), we fit a generalized mixed-effects model to examine whether children’s authority contingency judgments varied by norm type. The fixed-effects structure included Norm Type (Moral, Fairness, Conventional), and Participant ID was entered as a random intercept in the model. Although the Authority Contingency question was also asked in the Control scenarios, these were excluded from the model because relatively few children were asked it (it was only asked when a child said an act was not okay), and their inclusion did not alter the pattern of results.

Finally, for Forced-Choice Categorization, we used chi-square goodness-of-fit tests to examine whether children grouped Fairness scenarios with Moral or Conventional violations significantly different from chance.

## 3. Results

Data and analytic code for this project are openly available on the Open Science Framework (https://osf.io/jr2tk, accessed on 14 April 2026).

### 3.1. Act Evaluation

**Figure 3:**
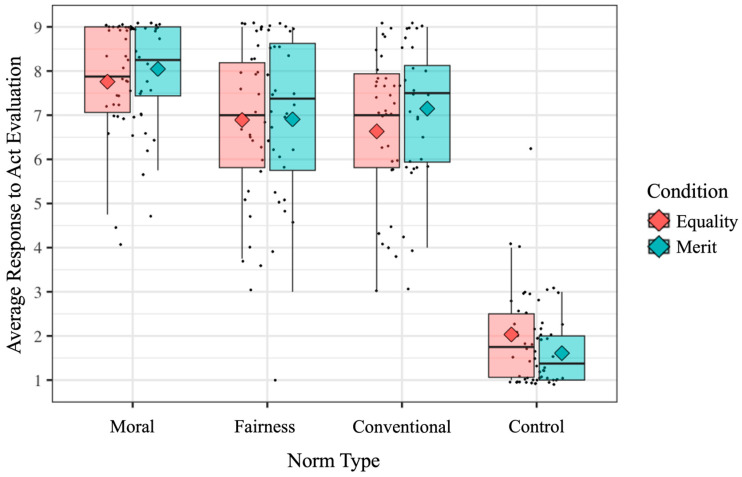
Children’s average responses to the act evaluation item (ranging from 1 = very, very nice to 9 = very, very bad) by condition (Equality and Merit) and Norm Type (Moral, Fairness, Conventional, and Control).

Figure 3 shows the average act evaluation of each scenario by Norm Type and Condition (Equality, Merit) (see Table A2 in Appendix B for means and standard deviations).

Because the design included repeated observations nested within children, we used mixed-effects models with participant entered as a random effect (see Section 2.4). This approach allowed us to retain all trial-level responses, account for within-subject dependence, and accommodate occasional missing item-level data. Our main question concerned whether children’s evaluations of fairness violations would differ depending on whether they were Equality or Merit violations. Contrary to our expectations, children in Merit (EMM = 6.91, SE = 0.23, 95% CI [6.46, 7.35]) and Equality conditions (EMM = 6.89, SE = 0.23, 95% CI [6.43, 7.35]) judged fairness violations to be similarly negative, *t*(247) = −0.05, *p* = 0.964. Further, as would be expected given that the other types of scenarios were identical across conditions, children did not differ across conditions in their evaluations of the Moral violations (*t*(247) = −0.89, *p* = 0.374), Conventional violations (*t*(247) = −1.59, *p* = 0.113), or Control scenarios (*t*(247) = 1.31, *p* = 0.192; all adjusted for multiple comparisons, Holm, 1978).

Next, we analyzed children’s responses within each condition separately. The act evaluation findings in the Equality condition replicated prior work (Yucel et al., 2022; Yucel & Vaish, 2026): 4-year-old children rated moral violations as more serious than Fairness-Equality violations, *t_MoralFairness_*(924) = 3.15, *p* = 0.003, and more serious than Conventional violations, *t_MoralConventional_*(924) = 4.08, *p* < 0.001. However, they rated the latter two as equal in seriousness *t_FairnessConventional_*(924) = 0.94, *p* = 0.349. Finally, as expected, all three types of violations (Moral, Fairness-Equality, and Conventional) were evaluated as worse than the Control scenarios, *t_MoralControl_*(924) = 20.78, *p* < 0.001, *t_FairnessControl_*(924) = 17.64, *p* < 0.001, and *t_ConventionalControl_*(924) = 16.70, *p* < 0.001.

Importantly, children’s evaluations of the violations in Merit condition also showed the same pattern as in the Equality condition: 4-year-old children rated Moral violations as more serious than Fairness-Merit violations, *t_MoralFairness_*(924) = 4.28, *p* < 0.001, and more serious than Conventional violations, *t_MoralConventional_*(924) = 3.37, *p* = 0.002. However, they rated the latter two as equal in seriousness *t_FairnessConventional_*(924) = −0.91, *p* = 0.364. Finally, as expected, all three types of violations (Moral, Fairness-Merit, and Conventional) were evaluated as worse than the Control scenarios, *t_MoralControl_*(924) = 24.13, *p* < 0.001, *t_FairnessControl_*(924) = 19.86, *p* < 0.001, and *t_ConventionalControl_*(924) = 20.77, *p* < 0.001.

### 3.2. Authority Contingency (Equality Condition Only)

Using a generalized mixed-effects logistic regression model, we examined whether children’s Authority Contingency judgments (0 = “Not okay,” 1 = “Okay if the teacher says it’s okay”) differed by norm type (Moral, Fairness-Equality, Conventional). Consistent with Yucel et al.’s (2022) Study 1 results with 4-year-olds, we found that across all three violation types, most 4-year-old children said the act would still not be okay even if the teacher allowed it (Fairness-Equality: 68.22% “not okay”; Conventional: 64.42%; Moral: 70.18%), and norm type did not reliably predict these judgments, χ^2^(2) = 3.05, *p* = 0.218. Relative to Fairness-Equality violations, children were not significantly more or less likely to say the Conventional violations (*b* = 0.63, *SE* = 0.49, z = 1.27, *p* = 0.203) or the Moral violations would be okay if the teacher allowed it (*b* = −0.19, *SE* = 0.50, *z* = −0.38, *p* = 0.706). A chi-square test of independence yielded a similar pattern of results, *χ*^2^(*df* = 2) = 0.84, *p* = 0.656.

### 3.3. Forced-Choice Grouping

When asked to choose whether the four fairness scenarios (as a group) belonged with the four Moral or four Conventional scenarios, 4-year-old children across both conditions showed no systematic patterns in their categorization. In the Equality condition, 13 of 30 children (43.33%) grouped Fairness-Equality scenarios with Moral scenarios, *χ*^2^(1) = 0.53, *p* = 0.465, replicating Yucel et al. (2022) and Yucel and Vaish (2026). Similarly, in the Merit condition, 15 of 32 children (46.88%) grouped the Fairness-Merit scenarios with Moral scenarios, *χ*^2^(1) = 0.13, *p* = 0.724.

Next, we sought to capture whether children gave more “normative” reasons, “emotional” reasons, or a combination of the two (or no reason at all) when categorizing unfairness as a moral or conventional violation. However, similar to Yucel et al. (2022) and Yucel and Vaish (2026), most children (Merit: 71.88%, Equality: 60%) did not provide any justification (see Table A1 in Appendix B). A handful of children gave norm-based justifications (Merit: 7 children, Equality: 3 children) or emotion-based justifications (Merit: 5 children, Equality: 5 children). Only one child in the Merit condition and none of the children in the Equality condition provided a mixture of normative and emotional explanations.

## 4. Discussion

Sensitivity to unfairness emerges early and remains central to how people think about deservingness and justice across the lifespan (Baumard et al., 2012; Dietze & Craig, 2021; Kanngiesser & Warneken, 2012; Schäfer et al., 2023; Sloane et al., 2012). Yet, how children conceptualize such violations—whether as moral wrongs akin to harm or as less serious conventional violations—remains unclear. The present study examined how 4-year-olds in the U.S. conceptualize two forms of distributional unfairness within the moral-conventional framework: simple inequality with no additional context (Fairness-Equality) and merit-based inequality in which equal effort was not matched by equal outcomes (Fairness-Merit). Using a paradigm adapted from prior work (Yucel et al., 2022; Yucel & Vaish, 2026), we replicated prior findings showing that equality-based unfairness occupies an ambiguous position between moral and conventional violations. Contrary to our predictions, however, adding merit information to highlight the unfairness of the outcome did not shift children’s judgments toward the moral domain.

Across conditions, 4-year-olds treated harm-based moral violations (e.g., hitting) as more serious than both equality-based and merit-based fairness violations, and more serious than conventional violations. Moreover, children evaluated both types of fairness violations (Equality and Merit) and conventional violations to be similar in seriousness, and as clearly worse than neutral control actions. Thus, by age 4, children may already see fairness violations as normatively impermissible, but they do not treat them as seriously as prototypical moral harm. This pattern fits with recent claims that children and adults may not perceive distributional unfairness to be equivalent to harm-based moral transgressions (Deutchman et al., 2025; Yucel et al., 2022; Yucel & Vaish, 2026).

### 4.1. Why Didn’t Effort “Moralize” Unfairness?

Critically, we found no difference between how children evaluated the simple inequality and merit-based inequality scenarios. Contrary to our prediction that highlighting equal effort would make the unequal outcome seem more severe and moral-like, children judged both types of unfairness as equally serious, and the overall pattern of evaluations of moral, fairness, conventional, and control scenarios was very similar across conditions. This contrasts with Yucel and Vaish (2026), who found that highlighting the harm caused by unfairness *did* shift both children’s and adults’ severity judgments toward the moral domain. Why did merit information fail to have a similar effect? We see two related possibilities.

First, harm salience and merit information may operate through different psychological pathways. One reason making harm salient may be particularly effective is that it addresses a core challenge in recognizing fairness as a moral violation: Unlike prototypical moral violations whose harm is direct and immediately visible, the harm caused by unfairness is often indirect and less perceptible (Ball et al., 2017; Yucel et al., 2022). By highlighting the victim’s harmed state (e.g., noting that the victim is sad), harm salience makes the otherwise imperceptible harm visible and concrete, helping children to identify a victim and represent the situation as interpersonal wrongdoing (see also Conry-Murray et al., 2025). Effort cues, by contrast, provide descriptive input about the allocation and deservingness (who “earned” what) but do not make the harm itself more direct or perceptible. They may therefore be less likely to change the perceived moral status of the act.

Second, even when children recognize something as unfair, effort information may lack the affective salience needed to shift moral judgments at this age. The emotional component of harm cues may operate as an attentional “spotlight” (Yucel & Westgate, 2021), amplifying moral considerations and increasing the perceived wrongfulness of the act. Effort information, being less emotionally evocative, may not produce a comparable shift in attention or moral evaluation. Without this affective amplification, merit-based violations may not be perceived as substantively different from equality-based violations: Both may be recognized as unfair, but neither elicits the empathic response that prototypical moral violations do (Hoffman, 2000; Vaish et al., 2009).

### 4.2. Limited Domain Differentiation in Authority Contingency and Categorization

The Authority Contingency and Forced-Choice Categorization tasks were intended to provide diagnostic judgments beyond evaluations of seriousness. Instead, both measures yielded minimal differentiation. On Authority Contingency, children treated moral, conventional, and fairness violations as similarly authority-independent: Across all three violation types, most children said the act would still not be okay even if a teacher said it was okay. This pattern replicates Yucel et al.’s (2022) Study 1 findings with 4-year-olds, and contrasts with classic Social Domain Theory findings that children judge moral norms as authority-independent and conventional norms as more authority-dependent (e.g., Smetana et al., 2012).

Here, it is important to note that Authority Contingency questions were included in the Equality condition but not the Merit condition. This was intended to keep the design of the Merit condition similar to the Harm Salience condition in Yucel and Vaish’s (2026) design, thereby allowing for closer comparisons between the effects of merit information and harm salience on children’s evaluations. We acknowledge, however, that this limits our ability to directly compare domain differentiation across the two conditions in the present study. Future research should address this asymmetry by incorporating Authority Contingency measures across all conditions.

Similarly, in the Forced-Choice Categorization task, children categorized fairness scenarios at chance, and most provided no explicit justification for their choices. These null findings are difficult to interpret, but in conjunction with similar results reported by Yucel et al. (2022), one interpretation is that 4-year-olds may not yet robustly differentiate fairness violations, even merit-based ones, using the criteria that distinguish moral from conventional norms. However, another possibility is that the forced-choice paradigm may not fully capture children’s conceptualization of fairness. If children view fairness as distinct from both moral and conventional domains—as some theoretical accounts and our findings suggest—then requiring them to choose between these two categories may obscure their underlying representations. Future research could address this limitation by using alternative task formats that allow children to treat fairness as a separate category and perhaps even differentiate between multiple fairness principles (e.g., equality, merit, and need).

### 4.3. The Ambiguous Status of Fairness Violations

Children in our study judged both equality-based and merit-based unfairness as “bad,” but perceived both to be about as serious as conventional violations and significantly less serious than prototypical moral violations, and showed no consistent pattern in categorizing them with moral or conventional violations. These findings, along with prior work on early fairness reasoning (Baumard et al., 2012; Sloane et al., 2012; Smith et al., 2013), suggest that 4-year-olds are sensitive to unfair outcomes but have not yet differentiated fairness violations as being clearly part of the moral domain.

One possibility is that this reflects a developmental trajectory in which early fairness concerns start out relatively undifferentiated, and perhaps closer to conventional than moral concerns. They then become more clearly differentiated and structured with age, shaped in part by parental socialization and cultural narratives about equality, deservingness, and meritocracy (Kinnard et al., 2025). In later childhood and even adolescence, children’s responses to various sources of inequality are refined as they increasingly consider different and often simultaneous factors such as merit, need, and group membership (Rutland & Killen, 2017). Thus, fairness may become more moralized over the course of development as children gain a richer understanding of harm, need, deservingness, identity, and norms, among other concerns.

An alternative possibility is that fairness does not simply “mature” into the moral domain, but rather remains distinct from prototypical moral concerns even into adulthood (Deutchman et al., 2025). Whether fairness violations are understood and responded to as moral wrongs may depend critically on context, shifting with the salience of harm to the disadvantaged party (Yucel & Vaish, 2026), the stakes of the allocation, and the cultural or situational factors that make a particular violation feel more or less like a moral transgression. Indeed, prior work documenting cultural variation in children’s responses to equality- and merit-based unfairness suggests that cultural emphasis on merit may shape whether such violations take on moral weight (Angarita & Viciana, 2022; Blake et al., 2015; Engelmann et al., 2021; Schäfer et al., 2015). In cultures that place less emphasis on meritocracy than the U.S., children may be less likely to treat merit-based violations as morally serious, and treat them instead as more conventional-like. This remains a critical question for future work.

### 4.4. Limitations and Future Directions

Some limitations of our study point to important directions for future research. First, our scenarios involved relatively low-stakes, everyday resources (e.g., chalk), and the transgressor always left some resources for the other child. Children might respond differently to merit-based inequalities involving more consequential goods, all-or-none distributions, or situations in which the emotional impact on the disadvantaged child is more vividly depicted (as in the harm salience scenarios in Yucel & Vaish, 2026).

Second, our sample was restricted to 4-year-olds in the U.S. Given that cultural context likely shapes children’s developing understanding of fairness and merit (as noted above), research across diverse sociocultural contexts is needed to understand the generalizability and variability of our findings.

Third, we relied on children’s verbal justifications in the Forced-Choice Categorization task, which many children did not provide. Future work might combine similar paradigms with more sensitive indices of affective and bodily engagement, such as physiological arousal or changes in posture (Gerdemann et al., 2022; Yucel et al., 2020) to test whether merit-based violations elicit distinct emotional signatures even when explicit judgments do not clearly differentiate them from equality-based violations.

## 5. Conclusions

In sum, our findings contribute to a growing body of evidence suggesting that children’s (and even adults’) understanding of fairness is more nuanced than previously thought. Although young children recognize the seriousness of moral violations involving explicit harm, they seem less certain about how to categorize fairness norms, both equality- and merit-based. Emphasizing equal effort does not, at this age, appear to shift perceptions of fairness toward more prototypical moral norms. Instead, fairness appears to occupy a mixed and somewhat unsettled position in children’s normative landscape—one that may be gradually reshaped as they encounter more complex social situations, stronger emotional reactions to injustice, and cultural messages about what people “deserve” for their efforts.

## Data Availability

The original data presented in the study are openly available in Open Science Framework at https://osf.io/jr2tk (accessed on 14 April 2026).

## Appendix A. (Study Script)

### Appendix A.1. Scale Introduction

The child was told via a video, “Thanks for playing with me today! In this game, I’ll tell you some stories, and you’ll tell me if what happened is nice or bad. Now I have these smiley faces! The green smiley face is for when things are really nice or ok, like when we play with our favorite toys. The red frowny face is for when things are really bad or not ok, like when we don’t get to play with our favorite toys. The orange face in the middle is for when things are a little bit bad. And these numbers are for you to show me how nice or bad each story is. If something is very, very nice, choose 9. If something is a little bit nice, choose 7. If something is very, very bad, choose 1. If something is very bad, choose 3. If something is bad, choose 4. If something is a little bit bad, choose 6. It gets nicer as it goes from red 1 to green 9.”

Then, child was asked where they would point if something was:

“… very, very nice?”

“… a little bit nice?”

“… very, very bad?”

“… a little bit bad?”

The experimenter corrected the child if the child pointed at the wrong item.

### Appendix A.2. Forced Choice Grouping Introduction

The child was told via a pre-recorded audio clip: “Now, before we start with our game, I wanted you to practice one more thing. Let’s practice sorting pictures into groups. You are going to see a picture in the middle, and then you can choose the group it should go to! Then the child was asked to decide which group an apple and a chair belonged to: “Where should this apple go? With the fruit or with the furniture?” and “Where should this chair go? With the fruit or with the furniture?”

### Appendix A.3. Vignettes

Once the child correctly identified scale items, the experimenter continued the Qualtrics survey, which presented Moral, Conventional, Fairness (Fairness-Merit, Fairness-Equality), and Control videos (16 videos total) in a completely randomized order.

*Moral Hit:* “This child hit this child.”

*Moral Push:* “This child pushed this child.”

*Moral Pull:* “This child pulled this child’s hair.”

*Moral Tear:* “This child tore this child’s picture.”

*Conventional Toy:* “This child did not put away her toy during snack time.”

*Conventional Pajamas:* “This child wore pajamas to school.”

*Conventional Wrong place:* “This child sat at the wrong place.”

*Conventional Story:* “This child did not listen and walked away during story time.”

*Fairness-Merit Playdoh:* “These children got to play with playdoh after they both worked very hard on a project for school. This child took more playdoh than this child.”

*Fairness-Merit Chalk:* “These children both helped the same amount to bring the chalk outside. During recess, this child took more chalk than this child.”

*Fairness-Merit White crayon:* “These children worked equally to reach the crayons from the top shelf. This child took all the colored crayons, leaving a white crayon for the other child.”

*Fairness-Merit Cookies:* “These children worked the same amount to bake cookies. This child took three cookies, leaving one cookie for this child.”

*Fairness-Equality Playdoh:* “This child took more playdoh than this child.”

*Fairness-Equality Chalk:* “This child took more chalk than this child.”

*Fairness-Equality White crayon:* “This child took all the colored crayons, leaving a white crayon for the other child.”

*Fairness-Equality Cookies:* “This child took three cookies, leaving one cookie for this child.”

*Control Thank you:* “This child said thank you after someone gave her a toy.”

*Control Permission:* “This child asked for permission to play with a toy.”

*Control Eat:* “This child sat down to eat with this child.”

*Control Play:* “This child played at the playground.”

### Appendix A.4. Act Evaluation

After each action, the video asked, “How nice or bad is that?” If the child did not respond, the children heard, “Could you show on this [scale]?” After each action was rated, the experimenter progressed to the next page.

### Appendix A.5. Authority Contingency (Fairness-Equality Only)

If a participant responded that any of the acts was bad, children were asked, “If your teacher said it was okay to do this, would it be okay or not okay?”

### Appendix A.6. Forced Choice Grouping

Children then watched a video putting moral or conventional transgressions in one group on the screen one by one (order counterbalanced) after reiterating what happened in each vignette (e.g., “Remember these pictures? This child did not listen and walked away during story time. This child wore pajamas to school. This child sat at the wrong place. This child did not put away their toy during snack time. These are all in one group. They are all in the same group.”). The experimenter then presented the next video for the second group of transgressions (e.g., “Remember these pictures? This child hit this child. This child pushed this child. This child pulled the hair of this child. This child tore the picture that this child drew. These are all in one group. They are all in the same group.”).

Then, the experimenter showed the video of fairness transgressions (e.g., “Remember these pictures? This child took more playdoh than this child. This child took more chalk than this child. This child took almost all the colored crayons, leaving one crayon for the other child. This child took three cookies, leaving one cookie for this child.”) and asked, “Where should all these pictures go]? Should they go with this group [Moral] or this group [Conventional]? Go to the next page to choose!”

Once the child has made a choice, the pre-recorded audio clip asked, “Why do you think it should go here?” This concluded the study.

## Appendix B. Coding and Coder Agreement

Fairness-Merit. All parents consented to their session being recorded. Therefore, 32/32 children provided a recorded justification. Two coders had excellent agreement, *κ* = 0.93.

Fairness-Equality. The parents of two children did not want their session to be recorded. Therefore, 30/32 children provided a recorded justification. Two coders had excellent agreement, *κ* = 0.94.

**Table A1:** Coding Scheme (taken from Yucel et al., 2022) and the Percent of Children Who Used Each Type of Justification by Condition.

Justification Category	Definition	Fairness-Merit	Fairness-Equality
No Justification	Child did not respond, said “I don’t know,” or gave an unrelated answer, such as describing the pictures.	71.88%	60.00%
Normative	Child referred to how right/wrong or good/bad the actions were, e.g., “Because it’s bad stuff.”	9.38%	23.33%
Emotional	Child referred to actions being kind or mean, or to emotions, such as being hurt, e.g., “Other group really hurts other people’s feelings.”	15.63%	16.67%
Normative and Emotional	Child used both Normative and Emotional justifications.	3.13%	0.00%

**Table A2:** Means (and Standard Deviations) of Children’s Responses to Act Evaluation by Norm Type; Scale Ranged from 1 = Very, Very Nice to 9 = Very, Very Bad.

Norm Type	Fairness-Merit	Fairness-Equality
Moral	8.05 (1.96)	7.76 (2.05)
Fairness	6.91 (2.82)	6.89 (2.46)
Conventional	7.15 (2.46)	6.63 (2.71)
Control	1.61 (0.99)	2.03 (1.98)
